# Redetermination of tetra­methyl tetra­thia­fulvalene-2,3,6,7-tetra­carboxyl­ate

**DOI:** 10.1107/S1600536812029534

**Published:** 2012-07-07

**Authors:** Felix Katzsch, Edwin Weber

**Affiliations:** a Institut für Organische Chemie, TU Bergakademie Freiberg, Leipziger Strasse 29, D-09596 Freiberg/Sachsen, Germany

## Abstract

An improved crystal structure of the title compound, C_14_H_12_O_8_S_4_, is reported. The structure, previously solved using the heavy-atom method (*R* = 7.1%), has now been solved using direct methods. Due to the improved quality of the data set an *R* value of 2.06% could be achieved. In the crystal, C—H⋯S and C—H⋯O contacts link the mol­ecules.

## Related literature

For the first structure determination of the title compound, see: Belsky & Voet (1976 ▶). For a previously reported experimental procedure and physical data, see: Yoneda *et al.* (1978 ▶). For C—H⋯O hydrogen bonds, see: Desiraju & Steiner (1999 ▶); Katzsch *et al.* (2011 ▶); Fischer *et al.* (2011 ▶). For C—H⋯S hydrogen bonds, see: Mata *et al.* (2010 ▶); Novoa *et al.* (1995 ▶); Lu *et al.* (2005 ▶); Saad *et al.* (2010 ▶). For a description of ring motifs, see: Bernstein *et al.* (1995 ▶); Petersen *et al.* (2007 ▶). For several steps of the synthetic procedure, see: Degani *et al.* (1986 ▶); O’Connor & Jones (1970 ▶); Nguyen *et al.* (2010 ▶). For general background to the electroconductive behaviour of tetra­thia­fulvalene derivatives, see: Takase *et al.* (2011 ▶).
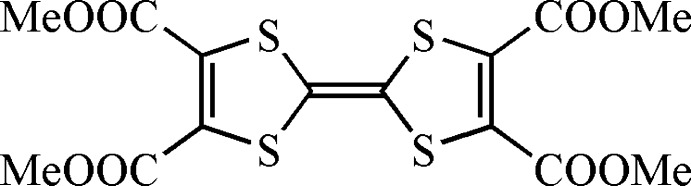


## Experimental

### 

#### Crystal data

C_14_H_12_O_8_S_4_
*M* *_r_* = 436.48
Triclinic, 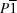

*a* = 6.8666 (2) Å
*b* = 7.8783 (2) Å
*c* = 8.4335 (2) Å
α = 100.221 (1)°
β = 99.255 (1)°
γ = 99.328 (1)°
*V* = 434.53 (2) Å^3^
*Z* = 1
Mo *K*α radiation
μ = 0.59 mm^−1^
*T* = 100 K
0.64 × 0.16 × 0.15 mm

#### Data collection

Bruker APEXII CCD area-detector diffractometer
Absorption correction: multi-scan (*SADABS*; Sheldrick, 2004 ▶) *T*_min_ = 0.705, *T*_max_ = 0.917
10884 measured reflections
1534 independent reflections
1471 reflections with *I* > 2σ(*I*)
*R*_int_ = 0.021

#### Refinement

*R*[*F* ^2^ > 2σ(*F* ^2^)] = 0.021
*wR*(*F* ^2^) = 0.055
*S* = 1.08
1534 reflections
120 parameters
H-atom parameters constrained
Δρ_max_ = 0.25 e Å^−3^
Δρ_min_ = −0.23 e Å^−3^

### 

Data collection: *APEX2* (Bruker, 2007 ▶); cell refinement: *SAINT* (Bruker, 2007 ▶); data reduction: *SAINT*; program(s) used to solve structure: *SHELXS97* (Sheldrick, 2008 ▶); program(s) used to refine structure: *SHELXL97* (Sheldrick, 2008 ▶); molecular graphics: *ORTEP-3* (Farrugia, 1997 ▶); software used to prepare material for publication: *SHELXTL* (Sheldrick, 2008 ▶).

## Supplementary Material

Crystal structure: contains datablock(s) I, global. DOI: 10.1107/S1600536812029534/im2387sup1.cif

Structure factors: contains datablock(s) I. DOI: 10.1107/S1600536812029534/im2387Isup2.hkl

Supplementary material file. DOI: 10.1107/S1600536812029534/im2387Isup3.cml

Additional supplementary materials:  crystallographic information; 3D view; checkCIF report

## Figures and Tables

**Table 1:** Hydrogen-bond geometry (Å, °)

*D*—H⋯*A*	*D*—H	H⋯*A*	*D*⋯*A*	*D*—H⋯*A*
C7—H7*A*⋯S1^i^	0.96	2.83	3.735 (2)	158
C5—H5*A*⋯O1^ii^	0.96	2.50	3.324 (2)	143
C5—H5*C*⋯O3^iii^	0.96	2.65	3.481 (2)	145

Symmetry codes: (i) 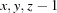; (ii) 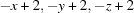; (iii) 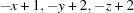.

**Fig. 1.**
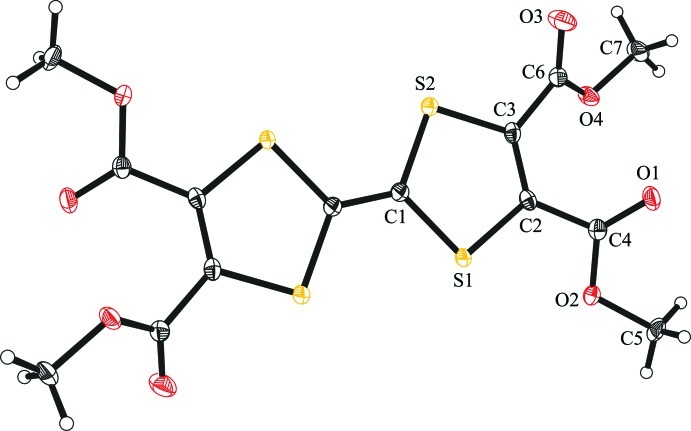
Perspective view of the title compound showing thermal ellipsoids at the 50% probability level.

**Fig. 2.**
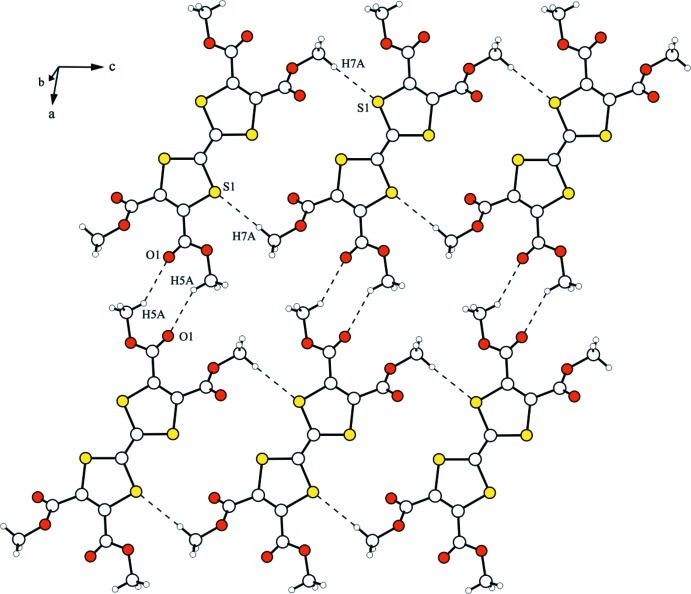
Hydrogen-bonds within the layer structure of tetra-substituted TTF.

## supplementary crystallographic information

### Comment

Tetrathiafulvalene derivatives are molecules of high importance relating to electroconductive behaviour (Takase *et al.*, 2011).

The present structure of the title compound (TTF) has been refined to an *R*-value of 2.06% which is clearly better than 7.1% of the previous study reported in 1976 by Belsky and Voet. In particular, this enabled us to refine the positions of hydrogen atoms of the methyl groups with improved accuracy making it possible to find potential hydrogen bonds. In conformity with previous findings, the TTF scaffold is planar and the methoxycarbonyl functions are slightly twisted out of the ring plane [interplanar angles 25.60 (1) and 42.77 (2)°] (Fig. 1).

The refinement shows the molecules being arranged in a layered structure (Fig. 2) stabilized by C—H···O (Desiraju *et al.*, 1999; Katzsch *et al.*, 2011; Fischer *et al.*, 2011) and C—H···S contacts (Mata *et al.*, 2010; Novoa *et al.*, 1995) (Table 1). Two molecules each are associated forming special dimer type species (Fig. 2). In one case, they involve two ester functions [*d*(C5—H5A···O1) = 2.50 Å] giving rise to a hydrogen bonded ring motif *R*^2^_2_(10) (Bernstein *et al.*, 1995; Petersen *et al.*, 2007). In the other case, adjacent molecules show hydrogen bonding interactions between ester methyl groups and sulfur atoms [*d*(C7—H7A···S1) = 2.83 Å] (Saad *et al.*, 2010; Lu *et al.*, 2005). The layers are also connected *via* C—H···O contacts including a methyl group and a carbonyl function [*d*(C5—H5C···O3) = 2.65 Å] of superimposed molecules.

### Experimental

The titled tetramethyl tetrathiafulvalene-2,3,6,7-tetracarboxylate was synthesized *via* a four step reaction sequence: (1) 1,3-Dithiolane-2-thione was prepared from carbon disulfide, sodium sulfide and 1,2-dichloroethane under phase transfer catalyzed condition following literature protocol (I. Degani *et al.*, 1986). (2) Reflux of 1,3-dithiolane-2-thione and dimethyl acetylenedicarboxylate in toluene yielded dimethyl 1,3-dithiole-2-thione-4,5-dicarboxylate (O'Connor & Jones, 1970). (3) This latter compound was treated with mercury(II) acetate in acetic acid/chloroform to obtain dimethyl 1,3-dithiole-2-one-4,5-dicarboxylate (Nguyen *et al.*, 2010). (4) In the final step, the 1,3-dithiol-2-one compound was coupled by a trimethyl phosphite induced reaction according to a literature protocol (Nguyen *et al.*, 2010). For this purpose methyl 1,3-dithiol-2-one-4,5-dicarboxylate (3.00 g, 12.8 mmol) was dissolved in trimethyl phosphite (7.94 g, 64.0 mmol) and stirred for 8 h at 100 °C. After cooling, a fine red precipitate had formed which was filtered and washed with a small amount of cold ethanol to yield 1.62 g (58 %) of the substituted TTF. Physical data of the compound correspond to reported values (Yoneda *et al.*, 1978). Suitable dark red single crystals for X-ray diffraction were grown by slow evaporation from a solution of the title compound in chloroform.

### Refinement

H atoms were positioned geometrically and allowed to ride on their parent atoms, with C—H = 0.96 Å, and *U*_iso_=1.5 *U*_eq_ (parent atom).

### Crystal data

**Table:**

C_14_H_12_O_8_S_4_	*Z* = 1
*M**_r_* = 436.48	*F*(000) = 224
Triclinic, *P*1	*D*_x_ = 1.668 Mg m^−^^3^
Hall symbol: -P 1	Mo *K*α radiation, λ = 0.71073 Å
*a* = 6.8666 (2) Å	Cell parameters from 9962 reflections
*b* = 7.8783 (2) Å	θ = 2.5–45.3°
*c* = 8.4335 (2) Å	µ = 0.59 mm^−^^1^
α = 100.221 (1)°	*T* = 100 K
β = 99.255 (1)°	Needle, red
γ = 99.328 (1)°	0.64 × 0.16 × 0.15 mm
*V* = 434.53 (2) Å^3^	

### Data collection

**Table:**

Bruker APEXII CCD area-detector diffractometer	1534 independent reflections
Radiation source: fine-focus sealed tube	1471 reflections with *I* > 2σ(*I*)
Graphite monochromator	*R*_int_ = 0.021
phi and ω scans	θ_max_ = 25.0°, θ_min_ = 2.5°
Absorption correction: multi-scan (*SADABS*; Sheldrick, 2004)	*h* = −7→8
*T*_min_ = 0.705, *T*_max_ = 0.917	*k* = −9→9
10884 measured reflections	*l* = −10→10

### Refinement

**Table:**

Refinement on *F*^2^	Primary atom site location: structure-invariant direct methods
Least-squares matrix: full	Secondary atom site location: difference Fourier map
*R*[*F*^2^ > 2σ(*F*^2^)] = 0.021	Hydrogen site location: inferred from neighbouring sites
*wR*(*F*^2^) = 0.055	H-atom parameters constrained
*S* = 1.08	*w* = 1/[σ^2^(*F*_o_^2^) + (0.0273*P*)^2^ + 0.222*P*] where *P* = (*F*_o_^2^ + 2*F*_c_^2^)/3
1534 reflections	(Δ/σ)_max_ = 0.026
120 parameters	Δρ_max_ = 0.25 e Å^−^^3^
0 restraints	Δρ_min_ = −0.23 e Å^−^^3^

### Special details

**Table:**

Geometry. All s.u.'s (except the s.u. in the dihedral angle between two l.s. planes) are estimated using the full covariance matrix. The cell s.u.'s are taken into account individually in the estimation of s.u.'s in distances, angles and torsion angles; correlations between s.u.'s in cell parameters are only used when they are defined by crystal symmetry. An approximate (isotropic) treatment of cell s.u.'s is used for estimating s.u.'s involving l.s. planes.
Refinement. Refinement of *F*^2^ against ALL reflections. The weighted *R*-factor *wR* and goodness of fit *S* are based on *F*^2^, conventional *R*-factors *R* are based on *F*, with *F* set to zero for negative *F*^2^. The threshold expression of *F*^2^ > 2σ(*F*^2^) is used only for calculating *R*-factors(gt) *etc*. and is not relevant to the choice of reflections for refinement. *R*-factors based on *F*^2^ are statistically about twice as large as those based on *F*, and *R*- factors based on ALL data will be even larger.

### Fractional atomic coordinates and isotropic or equivalent isotropic displacement parameters (Å^2^)

**Table:**

	*x*	*y*	*z*	*U*_iso_*/*U*_eq_	
S1	0.30978 (5)	0.63798 (4)	1.10656 (4)	0.01479 (11)	
S2	0.06589 (5)	0.56717 (4)	0.76932 (4)	0.01637 (11)	
O1	0.69629 (14)	0.93615 (13)	0.94082 (12)	0.0194 (2)	
O2	0.72198 (13)	0.79160 (12)	1.14884 (11)	0.0167 (2)	
O3	0.27914 (16)	0.78034 (15)	0.55357 (13)	0.0265 (3)	
O4	0.56544 (14)	0.69314 (13)	0.64417 (11)	0.0181 (2)	
C1	0.0779 (2)	0.54255 (17)	0.97428 (16)	0.0140 (3)	
C2	0.4249 (2)	0.71176 (17)	0.95464 (16)	0.0131 (3)	
C3	0.31610 (19)	0.67605 (17)	0.80137 (16)	0.0138 (3)	
C4	0.62881 (19)	0.82552 (17)	1.00955 (16)	0.0138 (3)	
C5	0.9200 (2)	0.90095 (19)	1.21844 (18)	0.0196 (3)	
H5A	1.0006	0.8996	1.1353	0.029*	
H5B	0.9840	0.8560	1.3075	0.029*	
H5C	0.9061	1.0194	1.2584	0.029*	
C6	0.3832 (2)	0.72501 (17)	0.65299 (16)	0.0153 (3)	
C7	0.6552 (2)	0.7573 (2)	0.51653 (17)	0.0226 (3)	
H7A	0.5790	0.6946	0.4108	0.034*	
H7B	0.7911	0.7391	0.5270	0.034*	
H7C	0.6548	0.8805	0.5272	0.034*	

### Atomic displacement parameters (Å^2^)

**Table:**

	*U*^11^	*U*^22^	*U*^33^	*U*^12^	*U*^13^	*U*^23^
S1	0.01005 (18)	0.02032 (19)	0.01379 (18)	−0.00123 (13)	0.00238 (12)	0.00683 (13)
S2	0.01129 (18)	0.02217 (19)	0.01518 (18)	−0.00117 (13)	0.00129 (13)	0.00788 (13)
O1	0.0162 (5)	0.0197 (5)	0.0233 (5)	−0.0007 (4)	0.0067 (4)	0.0087 (4)
O2	0.0108 (5)	0.0206 (5)	0.0168 (5)	−0.0020 (4)	0.0011 (4)	0.0055 (4)
O3	0.0216 (6)	0.0423 (7)	0.0225 (5)	0.0111 (5)	0.0064 (4)	0.0184 (5)
O4	0.0158 (5)	0.0265 (5)	0.0161 (5)	0.0054 (4)	0.0076 (4)	0.0096 (4)
C1	0.0116 (6)	0.0158 (6)	0.0147 (6)	0.0017 (5)	0.0017 (5)	0.0049 (5)
C2	0.0127 (6)	0.0131 (6)	0.0160 (6)	0.0035 (5)	0.0064 (5)	0.0053 (5)
C3	0.0109 (6)	0.0133 (6)	0.0184 (7)	0.0019 (5)	0.0046 (5)	0.0050 (5)
C4	0.0126 (6)	0.0143 (6)	0.0157 (6)	0.0035 (5)	0.0062 (5)	0.0022 (5)
C5	0.0104 (7)	0.0216 (7)	0.0231 (7)	−0.0023 (5)	0.0002 (5)	0.0024 (6)
C6	0.0154 (7)	0.0147 (6)	0.0152 (7)	0.0009 (5)	0.0032 (5)	0.0032 (5)
C7	0.0188 (7)	0.0342 (8)	0.0176 (7)	0.0022 (6)	0.0087 (6)	0.0110 (6)

### Geometric parameters (Å, º)

**Table:**

S1—C2	1.7452 (13)	C1—C1^i^	1.343 (3)
S1—C1	1.7570 (13)	C2—C3	1.3419 (19)
S2—C3	1.7468 (13)	C2—C4	1.4882 (18)
S2—C1	1.7636 (13)	C3—C6	1.4921 (18)
O1—C4	1.2022 (16)	C5—H5A	0.9600
O2—C4	1.3363 (16)	C5—H5B	0.9600
O2—C5	1.4559 (16)	C5—H5C	0.9600
O3—C6	1.2007 (17)	C7—H7A	0.9600
O4—C6	1.3263 (16)	C7—H7B	0.9600
O4—C7	1.4487 (16)	C7—H7C	0.9600
			
C2—S1—C1	94.90 (6)	O2—C5—H5A	109.5
C3—S2—C1	94.65 (6)	O2—C5—H5B	109.5
C4—O2—C5	115.25 (10)	H5A—C5—H5B	109.5
C6—O4—C7	115.57 (11)	O2—C5—H5C	109.5
C1^i^—C1—S1	122.42 (14)	H5A—C5—H5C	109.5
C1^i^—C1—S2	122.68 (14)	H5B—C5—H5C	109.5
S1—C1—S2	114.90 (7)	O3—C6—O4	125.74 (12)
C3—C2—C4	125.13 (12)	O3—C6—C3	122.87 (12)
C3—C2—S1	117.68 (10)	O4—C6—C3	111.34 (11)
C4—C2—S1	116.86 (10)	O4—C7—H7A	109.5
C2—C3—C6	126.96 (12)	O4—C7—H7B	109.5
C2—C3—S2	117.78 (10)	H7A—C7—H7B	109.5
C6—C3—S2	115.23 (10)	O4—C7—H7C	109.5
O1—C4—O2	125.14 (12)	H7A—C7—H7C	109.5
O1—C4—C2	124.48 (12)	H7B—C7—H7C	109.5
O2—C4—C2	110.33 (11)		
			
C2—S1—C1—C1^i^	178.58 (16)	C5—O2—C4—O1	0.21 (18)
C2—S1—C1—S2	−1.44 (8)	C5—O2—C4—C2	−177.38 (10)
C3—S2—C1—C1^i^	−177.53 (16)	C3—C2—C4—O1	22.1 (2)
C3—S2—C1—S1	2.49 (8)	S1—C2—C4—O1	−151.10 (11)
C1—S1—C2—C3	−0.67 (11)	C3—C2—C4—O2	−160.29 (12)
C1—S1—C2—C4	173.05 (10)	S1—C2—C4—O2	26.51 (14)
C4—C2—C3—C6	7.2 (2)	C7—O4—C6—O3	10.1 (2)
S1—C2—C3—C6	−179.69 (10)	C7—O4—C6—C3	−172.26 (11)
C4—C2—C3—S2	−170.52 (10)	C2—C3—C6—O3	−137.60 (15)
S1—C2—C3—S2	2.63 (15)	S2—C3—C6—O3	40.13 (17)
C1—S2—C3—C2	−3.06 (11)	C2—C3—C6—O4	44.73 (18)
C1—S2—C3—C6	178.99 (10)	S2—C3—C6—O4	−137.54 (10)

Symmetry code: (i) −*x*, −*y*+1, −*z*+2.

### Hydrogen-bond geometry (Å, º)

**Table:**

*D*—H···*A*	*D*—H	H···*A*	*D*···*A*	*D*—H···*A*
C7—H7*A*···S1^ii^	0.96	2.83	3.735 (2)	158
C5—H5*A*···O1^iii^	0.96	2.50	3.324 (2)	143
C5—H5*C*···O3^iv^	0.96	2.65	3.481 (2)	145

Symmetry codes: (ii) *x*, *y*, *z*−1; (iii) −*x*+2, −*y*+2, −*z*+2; (iv) −*x*+1, −*y*+2, −*z*+2.
